# Self-Assembly of Tunable Intrinsically Disordered Peptide Amphiphiles

**DOI:** 10.1021/acs.biomac.2c00866

**Published:** 2022-12-05

**Authors:** Tamara Ehm, Hila Shinar, Guy Jacoby, Sagi Meir, Gil Koren, Merav Segal Asher, Joanna Korpanty, Matthew P. Thompson, Nathan C. Gianneschi, Michael M. Kozlov, Salome Azoulay-Ginsburg, Roey J. Amir, Joachim O. Rädler, Roy Beck

**Affiliations:** † Raymond & Beverly Sackler School of Physics & Astronomy, Tel Aviv University, Tel Aviv 6997801, Israel; ‡ Faculty of Physics and Center for NanoScience, Ludwig-Maximilians-Universität, MünchenD-80539, Germany; § The Center for Physics & Chemistry of Living Systems, Tel Aviv University, Tel Aviv 6997801, Israel; ∥ The Center for NanoTechnology & NanoScience, Tel Aviv University, Tel Aviv 6997801, Israel; ⊥ Raymond & Beverly Sackler School of Chemistry, Tel Aviv University, Tel Aviv 6997801, Israel; # Department of Chemistry, International Institute for Nanotechnology, Chemistry of Life Processes Institute, Simpson Querrey Institute, Northwestern University, Evanston, Illinois 60208, United States; ∇ Department of Materials Science & Engineering, Department of Biomedical Engineering and Department of Pharmacology, Northwestern University, Evanston, Illinois 60208, United States; ○ Raymond & Beverly Sackler School of Medicine, Tel Aviv University, Tel Aviv 6997801, Israel; ◆ The ADAMA Center for Novel Delivery Systems in Crop Protection, Tel Aviv University, Tel Aviv 6997801, Israel

## Abstract

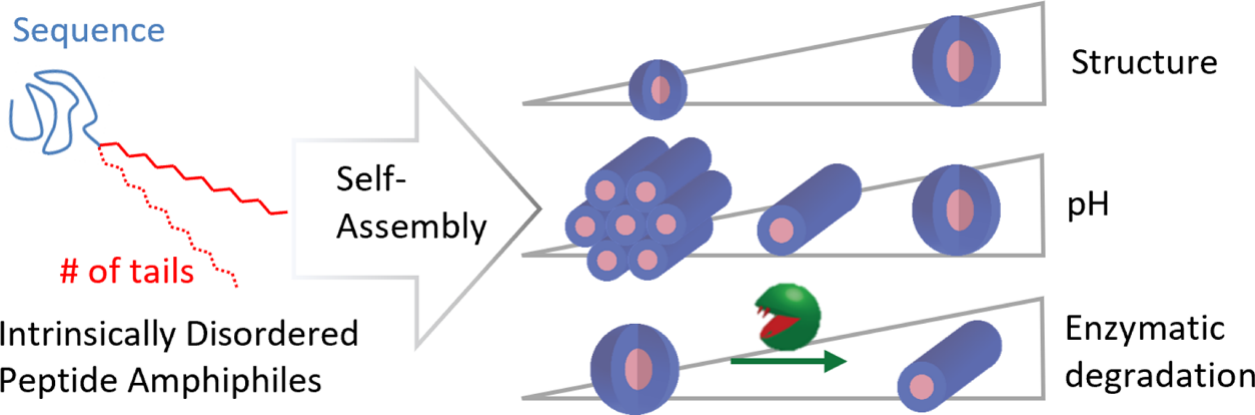

Intrinsically disordered peptide amphiphiles (IDPAs) present a novel class of synthetic conjugates that consist of short hydrophilic polypeptides anchored to hydrocarbon chains. These hybrid polymer-lipid block constructs spontaneously self-assemble into dispersed nanoscopic aggregates or ordered mesophases in aqueous solution due to hydrophobic interactions. Yet, the possible sequence variations and their influence on the self-assembly structures are vast and have hardly been explored. Here, we measure the nanoscopic self-assembled structures of four IDPA systems that differ by their amino acid sequence. We show that permutations in the charge pattern along the sequence remarkably alter the headgroup conformation and consequently alter the pH-triggered phase transitions between spherical, cylindrical micelles and hexagonal condensed phases. We demonstrate that even a single amino acid mutation is sufficient to tune structural transitions in the condensed IDPA mesophases, while peptide conformations remain unfolded and disordered. Furthermore, alteration of the peptide sequence can render IDPAs to become susceptible to enzymatic cleavage and induce enzymatically activated phase transitions. These results hold great potential for embedding multiple functionalities into lipid nanoparticle delivery systems by incorporating IDPAs with the desired properties.

## Introduction

Self-assembly of amphiphiles that combine hydrophilic and hydrophobic molecular moieties plays an omnipresent role in both natural and synthetic systems. In the biological world, lipid self-organization lies at the basis of cell membrane integrity, transport vehicles, and reaction vessels with precisely controlled size and functionality. In pharmacology, synthetic amphiphiles, in addition to natural lipids, are used to form nanoscopic carriers for encapsulating drugs.^1^ Following rational design principles, control of the size and stability of assemblies is achieved most prominently by using poly(ethylene glycol) (PEG)-lipid conjugates. These strategies result in highly efficient formulations such as lipid nanoparticles, which serve as RNA-based vaccine carriers against SARS-CoV-2,^2^ or other cargos or drugs.^3−9^ In order to advance the functionalities, nanocarriers composed of stimuli-responsive (e.g., enzymatic, pH, temperature) amphiphilic systems^10−16^ are studied, as they can potentially reduce the side effects of drugs by targeted release in tissues.

Amphiphiles can self-assemble into various mesophases in solution. Their mesoscopic morphology is, to a first approximation, determined by the volumetric ratio of the effective hydrophilic head group to the hydrophobic tail, as described by the so-called packing parameter.^17^ Here, the hydrophobic domain is composed of one or two fatty acid-based chains, as was previously demonstrated.^18^ In recent works, polypeptide chains have been conjugated to a hydrophobic domain to create peptide amphiphiles.^19−22^ In these studies, the polypeptides exhibited folded conformations and formed well-controlled nanoscale assemblies, such as long nanorods, that proved capable of encapsulating and releasing small molecules.^23,24^ The folded hydrophilic head group can lead to specific and relatively rigid structures that specific enzymes can recognize. Thus, these structures are potentially beneficial in applications where specific ligand-receptor binding is required.^25,26^

As in many other cases in biology, liquid-like structures dominated by weak and reversible interactions can be leveraged for novel biomedical applications. Indeed, and in contrast to the central dogma of protein folding, about half of the proteome contain proteins, and large domains that do not fold into rigid secondary or tertiary structures.^27−29^ These unfolded, intrinsically disordered proteins (IDPs) provide a significant functional advantage, enabling them to interact weakly with a broad range of binding partners, including themselves.^30,31^ Prominent examples of IDPs with weak interactions (i.e., on the order of thermal energy) include IDPs occurring in liquid-liquid phase separations^32^ or forming selective filters in nucleoporin complexes.^33^ Other examples of long disordered domains are the carboxy tails of intermediate filament proteins. These proteins retain their disordered nature even when constrained at high density^31,34,35^ and are responsible for fine-tuning the mechanical cytoskeleton behavior.^36−40^

Previous works showed that both the sequence composition and the fraction of charged amino acids play essential roles in the properties of a protein’s unfolded ensemble.^41,42^ For example, molecular dynamic simulations suggest that sequence composition and patterning are well reflected in the global conformational variables (e.g., the radius of gyration and the hydrodynamic radius), but end-to-end distance and dynamics are highly sequence-specific.^43^ Such analysis is suitable for comparing IDPs of different lengths.^29,44^ Moreover, it was demonstrated that the total net charge is inadequate as a descriptor of sequence–ensemble relationships for many IDPs. Instead, the sequence-specific distributions of oppositely charged residues are synergistic determinants of the conformational properties of polyampholytic IDPs.^45^

Sequence-encoded conformational properties can be extracted by calculating the charge patterning parameter (0 ≤ κ ≤ 1) and the fraction of charged residues (FCR).^45^ Low values of κ point to sequences where intrachain electrostatic repulsions and attractions are balanced. In contrast, high κ sequences show a preference for hairpin-like conformations caused by the long-range electrostatic attractions induced by conformational fluctuations.^45^ Other studies presented coarse-grain models that identify short-range electrostatic attractive domains between IDPs.^36,37,46^ Altogether, IDPs present an intriguing, unexplored territory that combines the structural plasticity of weakly interacting polymers with the specificity of the amino acid sequence.

In this context, intrinsically disordered peptide amphiphiles (IDPAs) are of great interest as they combine building blocks from natural lipids and proteins.^47−49^ IDPAs are composed of intrinsically disordered peptides conjugated to hydrocarbon chains, creating amphiphiles with polymeric headgroups and hydrophobic anchors that remain compatible with natural lipid membranes. Though IDPAs hold promise for fine-tuned nanoscopic self-assembly, the sequence space of even a 20 amino acid short polypeptide is extremely large and hardly explored.

Here, we present an approach to verify that structural transitions in IDPA assemblies depend on the peptide sequence, even though the headgroup conformation is disordered. We designed IDPAs with a peptide sequence inspired by a neurofilament low-chain protein and conjugated the sequence to a single or double hydrocarbon tail to compare peptides composed of the same amino acids but in a different sequence order. Using small-angle X-ray scattering (SAXS) and cryogenic transmission electron microscopy (cryo-TEM), we analyzed the nanoscopic structural phase transitions as a function of the pH and buffer salinity. We show that the phase transitions are controlled by the hydrophobic domain and charge pattern of the peptide sequence, which may induce hairpin-like conformations. Surprisingly, although the amphiphiles remain disordered, the mesoscopic structures exhibit low polydispersity. Structural phase transitions in mesoscopic order are sensitive to the mutation of a single amino acid in the polypeptide head group. Finally, we demonstrate that incorporation of suitable motifs renders IDPAs enzymatically cleavable. Ultimately, the reported sequence-dependent properties of IDPA mesophases could be exploited for the development of future drug carrier systems.

## Materials and Methods

### Synthesis and Purification

All peptides were synthesized via solid-phase synthesis and purchased from LifeTein. Amino acids are conjugated from the C-terminus to the N-terminus, while the peptide remains anchored to the insoluble solid resin support. The process involves repeated coupling cycles, washing, deprotection, and washing. The hydrophobic domain has either single or double hydrocarbon chains. After adding the last amino acid and deprotection, the fatty acid chain was conjugated to the deprotected amine. Double-chain PDAs were prepared by conjugation of Fmoc-Lys(Fmoc)-OH, followed by cleavage of the two Fmoc protecting groups and conjugation of the two tails.

### Sample Preparation

The IDPA or peptide powder was first fluidized in purified water (Milli-Q) at twice the desired concentration. The solution was then titrated with 1 M NaOH to a pH where the solution became more homogeneous (preferably a pH at which the IDPAs are soluble in water). Titration was monitored using a pH probe. Following titration, 50 μL of the solution was combined with 50 μL of a 2× buffer of choice to achieve a pH in the vicinity of the desired one. The 2× buffers acetic acid (pH 3-4.5), 2-(*N*-morpholino)ethanesulfonic acid (MES, pH 5-6.5), and 3-(*N*-morpholino)propane sulfonic acid (MOPS, pH 7–7.5), were prepared at 200 mM, to achieve a final buffer molarity of 100 mM after mixing with IDPA or peptide solution 1:1 (vol/vol).

### Circular Dichroism (CD)

Circular dichroism (CD) measurements were performed using a commercially available CD spectrometer (Applied Photophysics Chirascan). The IDPs were added to a glass cuvette with a 1 mm path length. The peptides were mixed with phosphate buffer to achieve a concentration of 0.1 mg/mL. The measurements were performed with phosphate buffer because the buffers used for the X-ray scattering experiments (mainly MOPS and MES) have high absorption at the relevant CD wavelengths. The wavelength range of 190–260 nm was measured in 1-nm steps with 0.5 s per point. Three measurements were performed for each sample, and the mean value was calculated.

### Computational Methods for Disorder Analysis

Disorder can also be analyzed computationally. IUPred2^50^ uses an energy estimation method. The principle lies in a 20 × 20 energy predictor matrix *P*_*ij*_ that shows the statistical potential for the 20 amino acids to connect with each other in a globular protein
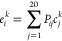
1where *e*_*i*_^*k*^ is the energy of the residue in position *k* of type *i*. The equation calculates for each position *k* the sum of all elements *j* in the amino acid composition vector *c*_*j*_ for all types *i*. The parameters are optimized to minimize the difference between energies estimated from the amino acid composition vector and the energies calculated from the known structure for each residue in the data set of proteins. As IUPred2, ANCHOR2^50^ also uses an energy estimation method and adds two more terms to the energy estimation: the interaction of the residues with the globular protein and the local environment. Thus, ANCHOR2 combines the disordering tendency calculated by Iurpred with the sensitivity to the environment of the protein and can predict if a specific region is disordered in isolation but can undergo disorder-to-order transition upon binding—without even knowing the possible binding partners. Netsurf 2.0^51^ is a sequence-based method and uses an architecture composed of convolutional and long short-term memory neural networks trained on solved protein structures to predict disorder.

### Cryo-TEM

Cryogenic TEM (cryo-TEM) specimens were prepared using an FEI Vitrobot by blotting in 95% humidity and subsequently plunging lacey carbon grids into liquid ethane. Images were taken for cryo-TEM using a JEOL 1230 transmission electron microscope operating at 120 keV equipped with a Gatan camera.

### Frequency Resonance Energy Transfer (FRET)

The fluorescence spectra of the IDPAs were measured using a Cary Eclipse fluorescence spectrophotometer (Agilent Technologies, Santa Clara, CA). Measurements were done in a 1 cm quartz cuvette at 10 μM concentrations in 100 mM buffer at 25°. The excitation spectra of IDP and IDPA included donor and acceptor (DA) spectra and acceptor only (AO) spectra. The samples were excited over the range of 250–330 nm (bandwidth 2.5 nm), and the emission was set to 350 nm (bandwidth 20.0 nm). The excitation spectra were normalized at 290–295 nm (no Tyr absorption). The level of energy transfer, E, between the donor and the acceptor, Y and W, respectively, was determined by the difference in integrated intensity at 270–285 nm and by using YW dipeptide as a reference for 100% energy transfer. Buffer and background signals were routinely measured and subtracted. The distance, r, was calculated using *E* = *R*_0_/(*R*_0_ + *r*), while the Forster radius, *R*_0_, was set as 15 Å.

### Small-Angle X-ray Scattering (SAXS)

All samples for SAXS were prepared at a final concentration of 5 mg/mL, which is an order of magnitude higher than the typical micro-molar CMC of 5 μM reported for similar peptide amphiphiles.^47,48,52,53^ For solubilizing conditions (above the transition pH, generally above pH 6), samples were measured at three synchrotron facilities: Beamline B21, Diamond Light Source, beamline SWING, SOLEIL synchrotron facility, Paris, France, and DESY, Hamburg, Germany. For phase-separating samples that display sediments (below the transition pH, generally pH 3–5.5), measurements were performed using an in-house X-ray scattering system, with a Genix3D (Xenocs) low-divergence Cu Kα radiation source (wavelength of λ = 1.54 Å) using a Pilatus 300K (Dectris) detector, as well as beamline I22 at Diamond Light Source. Samples were measured inside 1.5 mm quartz capillaries (Hilgenberg). All two-dimensional (2D) measurements were radially integrated using SAXSi^46^ to get one-dimensional (1D) intensity-scattering vector *q* data sets.

### Singular Value Decomposition (SVD)

In SVD, a minimum number of singular vectors represents the entire data set. Thus, these independent curves can represent the entire data set by their linear combinations
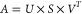
2where *U* yields a set of left singular vectors, i.e., orthonormal basic curves *U*(*k*) (*si*), that spans the range of matrix *A*. In contrast, the diagonal of *S* contains their associated singular values in descending order. For our scattering curves, the residuals are calculated via
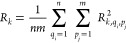
3where *m* is the size of the scattering vector *q* and *n* is the number of pH steps, and are plotted as a function of the number of singular vector components (*k*) that were chosen to reconstruct the data matrix. *R*_*k*,*q*_*i*_,*p*_*j*__ is defined by , where *D* is the data matrix, in which each column represents a one-dimensional scattering curve, *I*(*q*,*p*) at every pH step *p. D*_*k*_ is the reconstructed data matrix using *k* singular orthonormal vectors, and each term (*q*_*i*_, *p*_*j*_) in the matrix σ corresponds to the measured standard error for the corresponding term in *D*.

## Results

## IDPA Primary Structure

**Figure 1:**
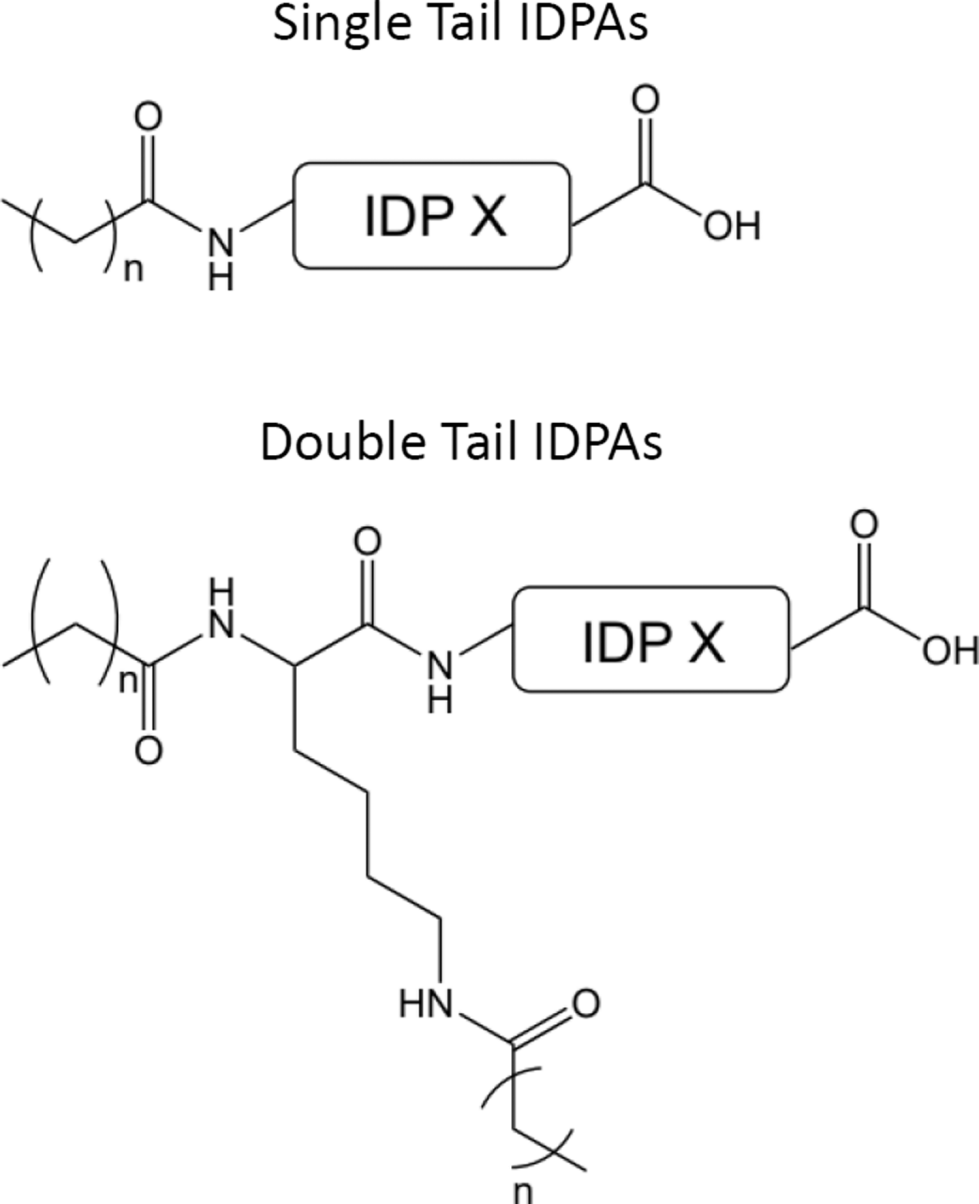
Chemical structures for double- and single-tailed IDPAs. For the detailed chemical structures of IDP X, see Supporting Figure S1.

In the present study, all IDPs are directly conjugated to fatty acids of various lengths to create the amphiphilic IDPAs. This study used various standard linear fatty acid chains with 12 (lauric acid), 14 (myristic acid), 16 (palmitic acid), and 18 (stearic acid) carbons (Table 1 for crucial parameters of IDPAs and Figure 1 for chemical structures). The IDPAs were synthesized using an automated solid-phase synthesizer. Thus, the molecular architectures are highly tunable, allowing us to study various hydrophobic and hydrophilic domains in a controlled manner. The peptide sequences are 18 amino acids long, containing protonable residues and hydrophilic amino acids (Supporting Figure S.1).

**Table 1:**
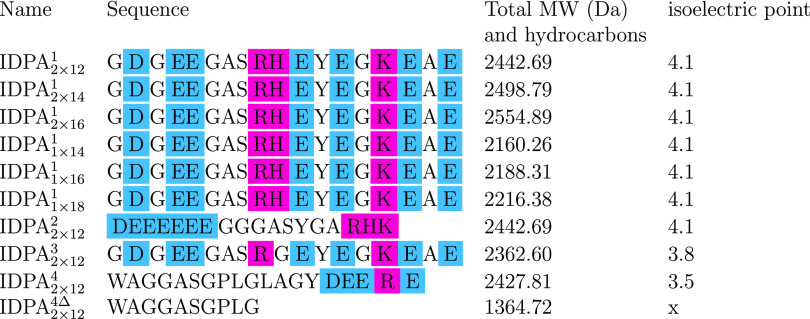
Key Parameters and Notations for the IDPAs Used in this Paper^a^

a The blue-colored letters stand for anionic amino acids and pink-colored ones for cationic amino acids. The upper case number in the IDPA name is the sequence number and the lower-case numbers are the number of tails in this molecule.

IDPA_2×12_^1^’s primary sequence (Table 1, Supporting Figure S.1) is inspired by the intrinsically disordered carboxy tail domain of neurofilament-light (NF-L) protein found in the cytoskeleton of nerve cells.^35−37,54^ In previous research,^47^ we introduced this IDP sequence to create IDPAs where aromatic branching units were used to cap the N-terminus of the IDP sequence and allow the branching into two different types of architectures containing either two or four hydrocarbon tails (2 × 12, 4 × 7, respectively). We showed that these IDPAs undergo a sharp phase transition from low-dispersity micellar spheres to extremely elongated worm-like micelles. Here, we present IDPAs that can be entirely prepared using conventional automated solid-phase peptide synthesis and allow us to study alternative molecular architectures in further depth. Inspired by the biological sequence of the NF-L protein, we synthesized the sequence IDP^1^ with various hydrocarbon tails. By slightly modifying this sequence, we studied how the interaction between the IDPs results in altered self-assembled structures once conjugated to the hydrophobic core.

IDPA^1^ series were synthesized with one or two aliphatic tails with three different tail lengths (1 × 14, 1 × 16, 1 × 18 and 2 × 12, 2 × 14, and 2 × 16) to investigate the influence of the hydrocarbon tail domain (Table 1, Supporting Figure S.1). In IDPA_2×12_^2^, we segregated the negatively charged amino acids at the N-terminus, while the positive ones were placed at the C-terminus. Hence, both IDPA_2×12_^1^ and IDPA_2×12_^2^ have identical magnitudes of net charge per residue (NCPR ≈ −0.278) at physiological pH.

Notably, the two peptide sequences include 11 chargeable residues, allowing for the net charge of the peptide to vary significantly as a function of pH. Electrostatic interactions are thus expected to play a significant role in the amphiphiles’ interactions and self-assembly. For both IDPAs, the isoelectric point (pI) is calculated at pH 4.1. At higher pHs, and in particular above pH 5.5, there is a decrease in the net charge to negative values due to the complete deprotonation of the aspartic acid and glutamic acid residues (Figure S.15).

To investigate the role of a single amino acid mutation, we designed IDP_2×12_^3^, where we replaced the positively charged histidine at position 10 of IDPA_2×12_^1^ with neutral glycine (Supporting Figure S.1), which decreases the isoelectric point to 4.0. In previous experiments, we found that the hydrophilic domain (i.e., the disordered peptide) and its interactions controlled the complex aggregations at low pH and served to strengthen the interaction between worm-like micelles.^47^ Here, we focus on the intermediate pH region where a single mutation can potentially fine-tune the phase transition point.

The peptides’ degree of disorder was experimentally verified by measuring the circular dichroism (CD) spectrum (Supporting Figure S.3, see Materials and Methods). In addition, the free peptides, IDP^1^, IDP^2^, and IDP^3^ were found to display a high probability for disorder and the absence of a regular secondary structure using Iupred/Anchor^50^ and NetSurf 2.0^51^ algorithm (Supporting Figures S.4–S.6; for analysis methods, see Materials and Methods). Interestingly, changing a single amino acid (His to Gly at position 10) from IDP^1^ to IDP^3^ changes the pH-dependent disorder. Specifically, in the vicinity of the isoelectric point, IDP^3^ bioinformation CD analysis indicates a possible ordering and lack of disorder, while IDP^1^ and IDP^2^ remain disordered throughout pH 2–10 (Supporting Figures S.4–S.6). Importantly, all of the bioinformatic analysis is conducted on peptide sequences alone, assuming they are a good proxy for the IDPAs that contain hydrophobic domains. We verified this assumption by measuring the frequency resonance energy transfer (FRET) of Tyr at position 14 and Trp at position 1 of IDP^4^ and IDPA_2×12_^4^. Here, we found no significant difference between the isolated peptide chain and when it is conjugated to the hydrophobic domain (Supporting Figure S.9; for FRET, see Materials and Methods).

### Amino Acids’ Charge Patterning Regulates the Self-Assembled Micellar Structure at High pH

The self-assembly of each IDPA was characterized by measuring the structural properties of pH-equilibrated samples using an in-house and synchrotron small-angle X-ray scattering (SAXS). SAXS allows direct evaluation in the solution of both the nanoscopic self-assembled structures and the mesophase symmetry (Supporting Information).

**Figure 2:**
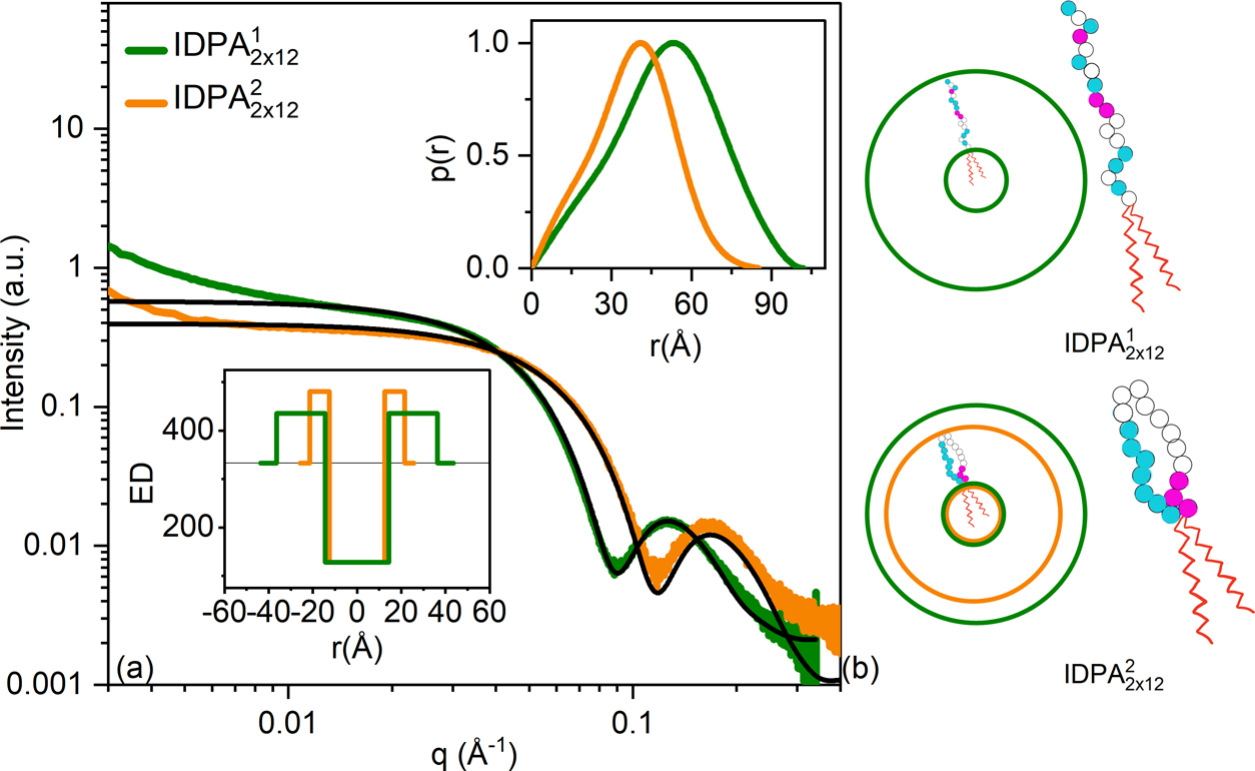
IDP head conformation as a function of sequence. (a) SAXS profiles show larger radii for IDPA_2×12_^1^ (green) than IDPA_2×12_^2^ (orange) at pH = 6.35 ± 0.5. Micellar core-shell form factor fits are shown in black line with the parameters detailed in Supporting Table S1. Lower inset: Electron density (ED) profile used in the fit. Upper inset: radius of gyration results of PDDF (*q*-range for fit: IDPA_2×12_^1^: 0.02–0.22 Å^–1^, IDPA_2×12_^2^: 0.02-0.24 Å^–1^). (b) Representation of the micellar sphere with IDPA_2×12_^1^ (green) and IDPA 2 (orange) with significantly different sizes of IDP layers and illustration of backfolding in IDPA_2×12_^2^ (lower cartoon) in comparison to IDPA_2×12_^1^ (upper cartoon). Pink circles indicate cationic, blue anionic, and white neutral amino acids.

We began our self-assembly investigation by comparing the structures for IDPA_2×12_^1^ and IDPA_2×12_^2^ at pH 6.5, both having 2 × 12 hydrocarbon chains. In such conditions, both IDPAs self-assemble into a dispersed micellar state but with shifted SAXS patterns (Figure 2). We fit the data using a spherical core-shell scattering form factor (Supporting eq 2) and find that IDPA_2×12_^2^ shows a significantly smaller radius (IDPA_2×12_^1^: 3.6 nm, IDPA_2×12_^2^: 2.1 nm). In addition, by extrapolating the form factor to zero momentum transfer, we find the aggregation number to be about 40 and 20 for IDPA_2×12_^1^ and IDPA_2×12_^2^, respectively. Given the similarity of the hydrophobic domain, the difference in radii thus originates from a smaller peptide layer (IDPA_2×12_^1^: 2.2 nm, IDPA_2×12_^2^: 0.9 nm, see Figure 2a lower inset). Pair distance distribution function (PDDF) evaluation^55^ confirms that the IDPA_2×12_^2^ micellar phase has a significantly smaller radius of gyration (Figure 2a, upper inset). Notably, the assembled structure at pH 6.5 is robust with low polydispersity, indicative of the sharp SAXS features.

### Phase Transitions Are Influenced by Charged Amino Acid Positioning

**Figure 3:**
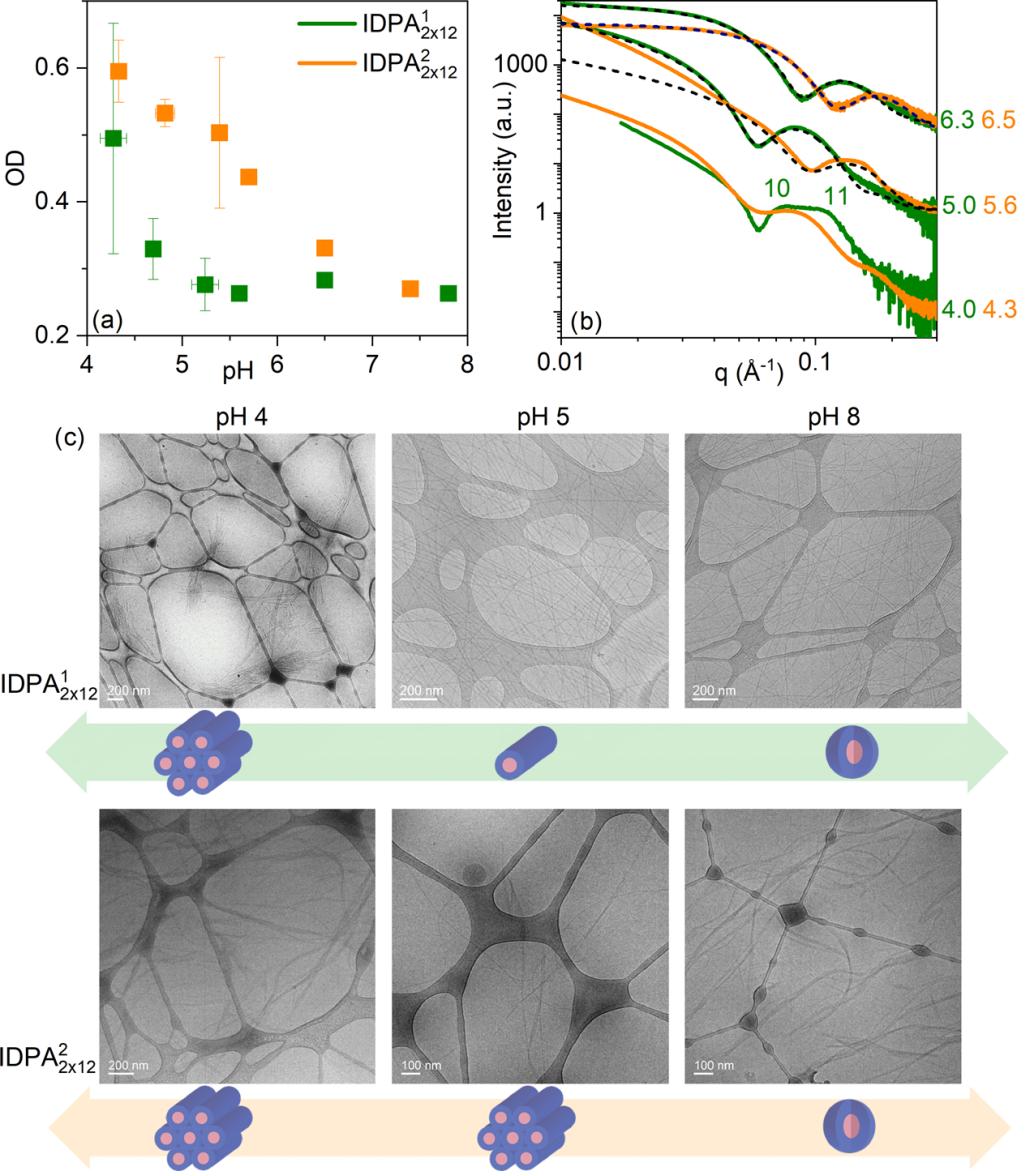
pH-dependent condensation of mesophases for IDPA_2×12_^1^ and IDPA_2×12_^2^—from bulk to dispersed phase. (a) Absorbance measurement shows high absorbance at the vicinity of the isoelectric point at pH 4.1; IDPA_2×12_^1^ shows a significantly milder slope than IDPA_2×12_^1^ during transitions between the two states. (b) SAXS scattering for IDPA_2×12_^1^ (green) and IDPA_2×12_^2^ (orange) at various pHs. Dotted lines show spherical/worm-like core-shell form factors. IDPA_2×12_^1^ at pH 4 shows humps that point toward a hexagonal phase. (c) Cryo-TEM pictures for IDPA_2×12_^1^ showing phase transition from spherical to worm-like micelles at pH 5. Aggregation of worm-like monodisperse micelles at the vicinity of the isoelectric point at pH 4.1.

Previous measurements^47^ of an amphiphile with a similar peptide head group as IDPA_2×12_^1^ showed that its self-assembled structure is pH-dependent due to changes in the charged amino acids. Here, we evaluate how charge patterning can tune the pH-dependent phase transitions for IDPA_2×12_^1^ and IDPA_2×12_^2^ using SAXS, turbidity measurements, and cryogenic transmission electron microscopy (cryo-TEM). IDPA_2×12_^1^ and IDPA_2×12_^1^ are insoluble close to the isoelectric point (pI). This indicates that peptide–peptide interactions are favored over peptide-water interactions.^56,57^ Away from the pI, the IDPAs become soluble and form monodisperse nanoparticles in the solution. These nanoparticles can be identified as spherical and/or cylindrical micelles using cryo-TEM and turbidity measurements (Figure 3). Furthermore, SAXS data analysis and cryo-TEM direct imaging reveal that micellar rods collapse into a condensed phase in the vicinity of the pI (Figure 3). For IDPA_2×12_^1^, the SAXS data points towards a hexagonal phase (Figure 3). This transition from worm-like monodisperse micelles to hexagonal packed ones was also studied by turbidity measurements, showing a clear optical difference between the condensed and dispersed phases. Specifically, while IDPA_2×12_^1^ transitions in a relatively small pH interval (pH 4.2–4.6), IDPA_2×12_^1^ shows a significantly wider range for the transition (pH 4.2–6.5).

#### Both Peptide Sequence and Hydrocarbon Chain Length Tune the Spherical to Rod-like Micelle Transition

**Figure 4:**
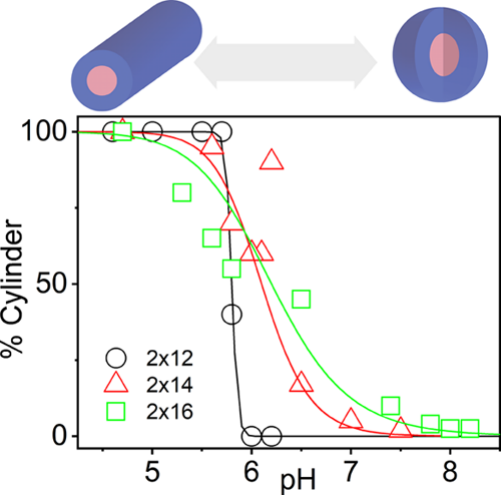
pH-dependent phase transition for different lipid chain lengths. The pH-dependent range of transition from worm-like (pH < 4.7) to spherical micelles (pH gt 7.5) broadens with increasing tail length (indicated in the legend). The phases in between are a superposition of form factors and are interpreted as coexistent. Lines represent a Hill function fit.

The balance between the architectures of the hydrophilic and hydrophobic domains plays a critical role in the self-assembly and phase transition of amphiphiles.^14^ Previously, we showed that hydrophobic dendritic domains conjugated to the peptide sequence of IDP^1^ could slightly alter the pH-induced phase transition from sphere to rod-like micelles.^47^ Here, we studied how the phase transition depends on the hydrocarbon length. Using SAXS, we find that double-chained IDPA_2×12_^1^ shows worm-like micelles at low pH and spherical micelles at high pH. At intermediate pH, we detect a coexistence regime with the combination of two mesophases by fitting the SAXS scattering through a linear combination of spherical and cylindrical core-shell shape factors (Figures 4, and Figure S.14). These results point to a continuous coexistence transition between spherical and worm-like micelles of constant radii. Significantly, the sharpness of the transition depends on the length of the tails: longer tails result in a phase transition at higher pHs with a much broader range (2 × 16: pH 4.7–7.8, 2 × 14: pH 4.7–7.5) between the two mesophases (Figure 4). On the contrary, the IDPA_2×12_^1^ with a shorter 2 × 12 tail transitions in a very narrow pH range (pH 5.7–6.0). Important to mention that for IDPA_2×12_^1^ the cylinders transition completely to spheres, whereas IDPA_2×14_^1^ and IDPA_2×14_^1^ have still a low fraction of cylinders (approx. 2%) at high pHs.

Using single-value decomposition (SVD, see Materials and Methods), we tested how many distinct scattering patterns contribute to the polydisperse signal for the transition pH range described before. We assume that the number of independent vectors resulting from the SVD analysis represents an upper bound to the number of different phases in the coexisting regime. Indeed, our IDPA transition requires up to 2–3 coexisting scattering vectors for the different IDPAs. Specifically, for IDPAs with tail lengths of 2 × 12 and 2 × 16, there are up to three different phases, and for IDPA_2×14_^1^, only two different phases are required by the SVD analysis (Figure S.10). This result also agrees well with our initial finding that the IDPA transitions from spheres to rods, and in between, we have a linear superposition of the two dominant form factors. For IDPA_2×12_^1^ and IDPA_2×16_^1^, we found that three independent vectors can describe the data. A possible explanation is an intermediate phase, e.g., an ellipsoidal phase, between the rod and the spherical phase that, unfortunately, is too weak for us to fit even by synchrotron’s SAXS data.

**Figure 5:**
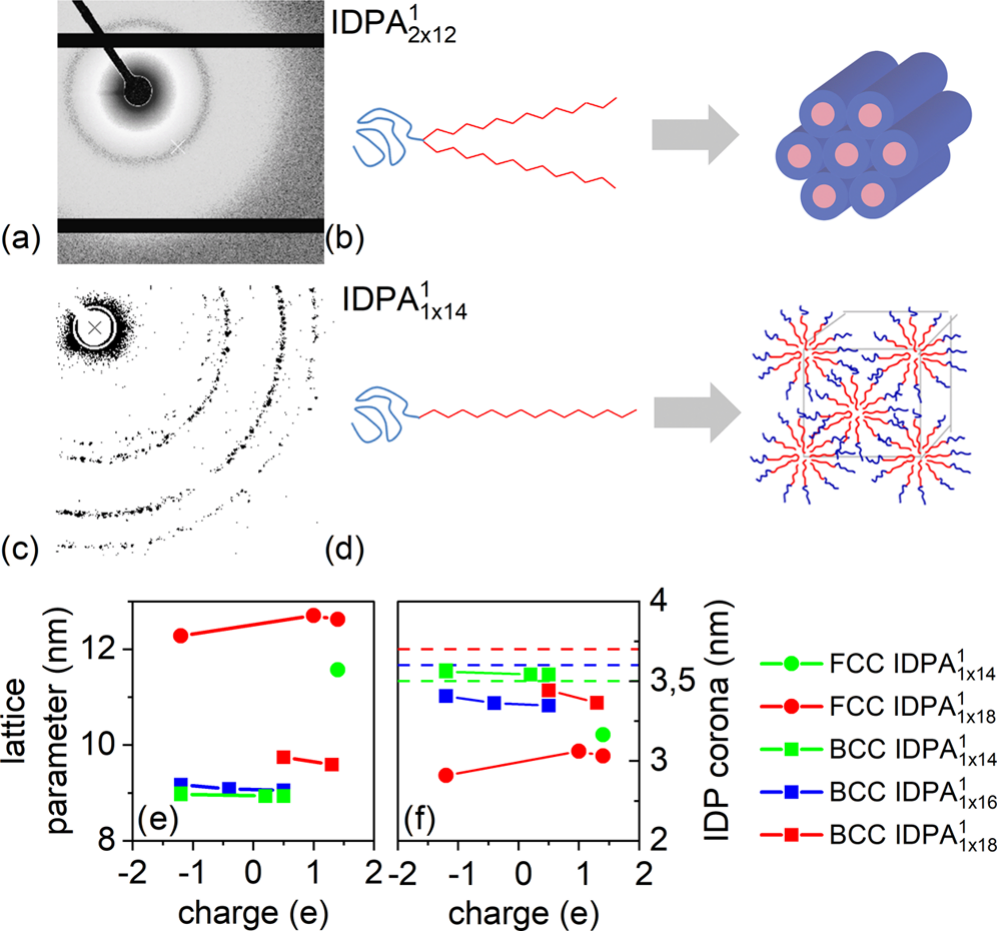
Formation of liquid crystals at isoelectric point (pH 4.3) for single-tailed IDPAs with different tail lengths. 2D SAXS pattern for (a) double- and (c) single-tailed IDPAs at isoelectric point (pH 4) showing hexagonal and FCC phases. (b) and (d) are related cartoons illustrating the formation of mesophases from the double- and single-tailed IDPAs, respectively. Lattice parameters (*d*) for (e) BCC and (f) FCC phases from integrated 1D patterns for single-tailed IDPAs near the isoelectric point were found by extracting the peak position via gaussian fit. The charge is calculated via summation of amino acids’ charges at various pHs. Unit cell dimensions are directly measured from SAXS correlation peak positions. Nearest neighbors (dashed lines) are extracted using *d*√2/2 for FCC and *d* for BCC. IDP headgroup layer sizes for IDPA_1×14_^1^, IDPA_1×16_^1^, and IDPA_1×18_^1^ are extracted by subtracting the calculated tail length (, see text) from the lattice parameter.

The number of hydrocarbon chains is another architectural feature when designing IDPAs. For double-chained IDPAs, the SAXS pattern is isotropic as the nanoparticles scatter in all possible orientations (Figure 5a). However, while IDPAs with single hydrocarbon tails (IDPA_1×14_^1^, IDPA_1×16_^1^, and IDPA_1×18_^1^) show isotropic micellar spheres at intermediate and high pHs, they collapse into liquid crystals with a strong “spackle” pattern close to the pI. The scattering peak positions indicate face-centered cubic (FCC) and body-centered cubic (BCC) Bravais lattices (Figure 5b). Importantly, around the pI, the FCC and BCC organizations and “spackle” scatterings are evidence of soft IDPA monodispersed micelles packed into rather large “crystals” on the incoming beam dimensions (≈1.5 mm^2^). The SAXS analysis reveals that the lattice parameters for both FCC and BCC are proportional to hydrocarbon tail lengths (, see Figure 5c,d). Using  as an approximation for hydrocarbon tail extension,^17^ we can extract the approximate size of the hydrophilic domain thickness to be around 2.7 nm. The hydrophilic domain thickness does not depend on the hydrocarbon tail length. Moreover, the IDP layer at the isoelectric point is in agreement with the IDP layer of micellar spheres fitted at intermediate pH (Supporting Table S.1 and Figure S.13 and dashed lines in Figure 5c,d).

After studying how the length and the number of tails affect the self-assembly of the IDPAs, we set to explore how minor alterations in the peptide sequence can tune the phase transition. For example, IDP_2×12_^3^, which is different from IPDA1 only by a single amino acid at position 10, transitions at pH 5.4 from spherical to cylindrical micelles, while the equivalent IDPA_2×12_^1^ transitions at pH 5.8 (Supporting Figure S.15). The altered transition can be attributed to differences in interactions resulting from exchanging histidine (*pK*_a_ = 6.0) with the neutral glycine. An alternative route to influence the self-assembly is through the introduction of salt (NaCl), which screens the electrostatic interactions between neighboring charged peptides. Using Kratky analysis on the SAXS data, we reveal the compactness of the IDPAs at varying salt concentrations (Supporting Figure S.11). We find a trend toward higher slopes in the high momentum vector (*q*) region with increasing salt concentration. This is more pronounced with increasing chain length. The high slope indicates that the IDPAs are more unfolded than at low salt concentrations. For the low *q*-region, the dispersity between the curves becomes more pronounced with increasing chain length.

### Cleavable IDPAs

One of the advantages of IDPAs is the ability to design sequences that can interact with other biological entities. For example, the utilization of IDP as the hydrophilic domain can be designed to interact with an enzyme, in order to induce drug release from the self-assembled nanocarrier or aggregation of the carrier at the site of enzymatic activation.^58,59^ Therefore, we designed the additional IDPA sequence (IDPA_2×12_^4^, Supporting Figure S.1) that contains a cleavage domain (GPLGLAG) for an MMP-9 enzyme. Indeed, upon incubation with the MMP-9 enzyme, the IDPA is cleaved with a shortened peptide sequence (Supporting Figure S.2). We term the remaining amphiphile, which includes the hydrophobic domain, as IDPA_2×12_^4Δ^ and the cleaved peptide as IDP^4δ^.

The cleavage site in IDPA_2×12_^4^ was introduced to dramatically disturb the self-assembled structure via enzymatic reaction. The sequence conjugated to the hydrocarbon (IDP^4Δ^) contains neutral amino acids. It is on the threshold of being disordered, while the remaining part (after the cleavage site), termed here IDP^4δ^, contains partially protonatable amino acids and is expected to be disordered at all pHs (Supporting Figures S.7 and S.8). Both IDPA_2×12_^4^ and IDPA_2×12_^4Δ^ were measured at various pHs, and their self-assembly was studied using SAXS.

**Figure 6:**
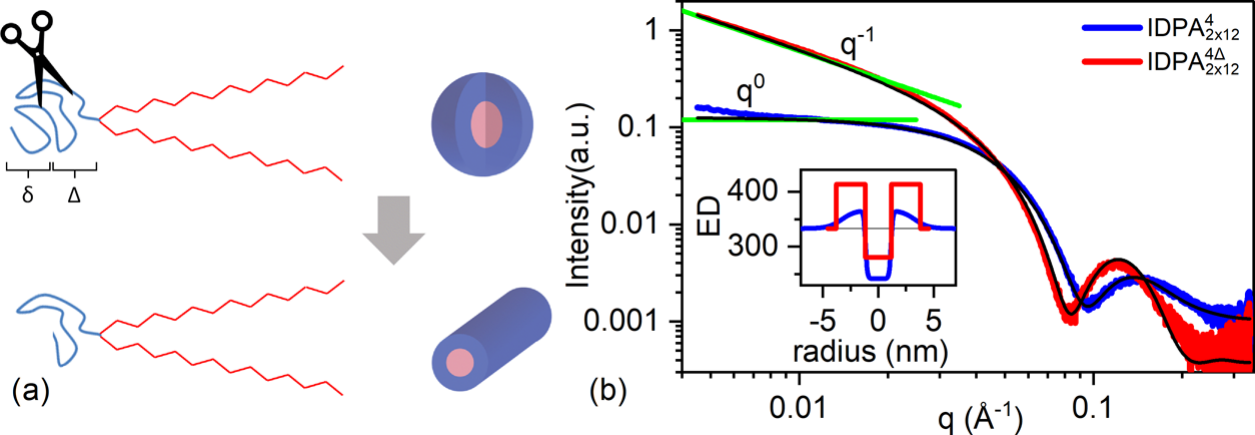
SAXS data for cleavable IDPA. (a) Cartoon showing the self-assembly of spherical and worm-like micelles for IDPA_2×12_^4^ and IDPA_2×12_^4Δ^, respectively. (b) SAXS data and fit for the IDPAs (blue IDPA_2×12_^4^, red IDPA_2×12_^4Δ^) at physiological pH (pH 7). Inset shows electron density (ED) profiles. Green lines show small-angle region fits used for initial structural determination.

At physiological pH, IDPA_2×12_^4^ assembles into spherical micelles, indicated through the scattering intensity at small angles,^60^*I*(*q* → 0) ∼ *q*^0^, while IDPA_2×12_^4Δ^ forms worm-like micelles with *I*(*q* → 0) ∼ *q*^–1^ (Figure 6a). We further fit the SAXS data using a (smooth) spherical core-shell model and a cylindrical core-shell model and found that the hydrocarbon domain stays constant while the peptide layer of the sphere smears toward higher radii with lower electron densities (Figure 6b).

At different pH values, IDPA_2×12_^4^ undergoes structural phase transitions while IDPA_2×12_^4Δ^ remains in a worm-like state (Supporting Figure S.16). In agreement with Takahashi et al.,^61^ the SAXS pattern for IDPA_2×12_^4^ at pH 5 indicates the formation of polymer vesicles upon stretching of spherical micelles.

Furthermore, this pH sensitivity was investigated by measuring the *R*_g_ versus the pH of the crude peptides using SAXS. The *R*_g_s for IDP^4^ and IDP^4Δ^ show little dependence on pH and are ∼9 and ∼11 Å, respectively. However, we assume that the *R*_g_ of IDP^4^ is more sensitive to pH (Figure S.17). This is indicative of the pH-sensitive phase change of IDPA_2×12_^4^ compared to IDPA_2×12_^4Δ^.

## Discussion

IDPAs present a highly modular molecular platform for the design of transformative nanocarriers.^47^ We presented new IDPA molecules, which were entirely synthesized by an automated solid-phase peptide synthesizer. A peptide sequence inspired by the disordered regions of the neurofilament-light chain protein was systematically altered to study how the interplay of hydrophobic tail(s) architecture and polypeptide headgroup conformation dictates the self-assembly process.

Despite sharing identical amino acids, IDPA_2×12_^1^ and IDPA_2×12_^2^, with similar hydrophobic domains, assemble into spherical micelles with different radii at high pH. Specifically, IDPA_2×12_^1^ assembly has a significantly larger polypeptide shell thickness than IDPA_2×12_^2^. This demonstrates how sequence ordering plays a dominant role in the assembly of IDPAs. In IDPA_2×12_^2^, we segregated the positively and negatively charged amino acids at the edges of the sequence. Therefore, the more compact peptide conformation is likely to result from transient backfolding of the peptide chains due to electrostatic interactions of the oppositely charged ends (Figure 2b).

Investigation of the self-assembly of the two IDPAs at different pH values revealed that the transition from a collapsed hexagonal phase at the isoelectric point to dispersed worm-like micelles is also sequence-dependent. For example, IDPA_2×12_^2^ transitions to a dispersed state over a relatively broad pH range compared to IDPA_2×12_^1^. Considering our previous results,^47^ we argue that the transient hairpin-shaped and more compact peptide conformation are less prone to interact with neighboring worm-like micelles. In a sense, for IDPA_2×12_^2^, the almost complete overlap between the peptides of opposing worm-like micelles is needed to induce electrostatic attraction, while for IDPA_2×12_^1^, only partial overlap is needed.

In addition, even a minor alteration, such as the exchange of a single amino acid in the peptide sequence, can tune the pH structural phase transition. Specifically, IDP_2×12_^3^ transitions between spheres to elongated worm-like micelles at pH 5.4, while IDPA_2×12_^1^ transitions at pH 5.8 with a change of one single amino acid (histidine to glycine). When calculating the net charge difference between IDPA_2×12_^1^ and IDP_2×12_^3^, one can expect that the phase transition will occur at pH 5.2 (Supporting Figure S.15a), although experimentally, the difference is milder. Using a free energy model for electrostatic repulsion contribution, we can explain this phenomenon.^47^ In short, the position of the charged amino acid along the polypeptide contributes to the electrostatic repulsion between the neighboring chains in proportion to their vicinity to the peptide-tail interface. Therefore, exchanging the charged histidine in the middle of the sequence has a relatively mild impact on the mesoscopic structural phase transition.

As an alternative means to alter the structural phase transition, we evaluated the role of the hydrophobic tail(s) domain. When introducing IDPAs with just one chain instead of two, the IDPAs self-assembled into large spherical micelle crystals close to the isoelectric point. As shown in Figure 5, the distance between these micelles within the crystals is significantly smaller than the micelles’ radii at slightly higher pHs. This indicates that the outer IDPs’ shells overlap between nearest neighbors. Such overlap is needed to induce short-ranged attractive forces between neighboring IDPAs, stabilizing the micellar crystals.

At intermediate pHs, the IDPAs are in the coexistence phase of spheres and cylinders, where the transition width broadens with increasing tail length. While a similar coexistence of rod and micellar phases, instead of elongated micelles with end caps, has been shown before,^62^ the correlation between the transition width and the chain length requires further explanation, as detailed below.

It was proposed that the reason for the coexistence between cylindrical micelles of finite lengths and spherical micelles is an energy barrier the system has to overcome on the way of transformation between the two types of micelles.^62^ This energy barrier originates from the difference between the energies of two end caps of a cylindrical micelle and the energy of a spherical micelle. Hence, such coexistence does not represent a thermodynamic equilibrium between the two phases, but rather indicates a slow transition between the two phases enabling simultaneous observation of both cylindrical and spherical micelles within the time scale of the experiments. In this model, the beginning of the coexistence region (Figure 4) corresponds to conditions upon which the energy barrier of formation of a spherical micelle out of a cylindrical one is such that the characteristic time of this event is comparable with the time of observation. At the end of the coexistence region, the energy barrier must be small enough to make the transition time shorter than the observation time. The origin of the energy barrier is an energetically unfavorable but unavoidable transition region, which builds up within a cylindrical micelle between its endcap and the cylindrical part because of a difference in their cross-sectional thicknesses.^62^ This difference results from packing molecules with a particular molecular volume and surface area into a spherical versus cylindrical aggregate. An increase in the spontaneous monolayer curvature driven by the charge growth at increasing pH makes the endcap more energetically favorable and hence decreases the energy barrier. A simple geometrical consideration explains that the shorter the IDPA chain length, the more minor the thickness mismatch between the endcap and the cylindrical part of a micelle and, therefore, the lower the initial energy barrier. As a result, less charge must be generated to cut down this energy barrier and facilitate a fast cylinder-to-sphere transition. This explains the chain length dependence of the width of the coexistence region (Figure 4).

We have shown that IDPAs can be engineered to induce phase transitions upon enzymatic activation. IDPA_2×12_^4^ self-assembles into spherical micelles, whereas upon enzymatic cleavage, the assembly of the cleaved IDPA_2×12_^4Δ^ transforms into worm-like micelles at physiological pH. Furthermore, we demonstrated that pH triggers phase transitions for the uncleaved peptide containing protonable amino acids, whereas pH does not affect the cleaved peptide containing only neutral amino acids. These results are of great interest for biomedical applications, given the ability to change the physical properties of the nanocarrier at constant pH by an enzymatic reaction. It thus suggests an alternative path for enzymatically triggered activation of drug release in a controllable manner. Furthermore, IDPA_2×12_^4^, in similarity to all other IDPAs presented here, shows remarkably controllable, monodisperse nanostructures. The pH dependency of IDPA_2×12_^4^ and IDPA_2×12_^4Δ^ self-assembly demonstrates the ability to design both pH-dependent and independent structures upon cleavage. Thus, our work enables us to combine enzymatic cleavage with pH-dependent phase transition in a single amphiphilic molecule.

## Conclusions

We have studied the self-assembly of five disordered polypeptide domains conjugated with different fatty acids in IDPAs. Even though polypeptide chain conformation is disordered, the interactions between the peptide headgroups lead to various distinct self-assembled nanostructures. The IDPA systems respond to pH and salinity and exhibit structural phase transitions depending on the peptide sequence and the number and length of the hydrocarbon tail.

It stands to reason that IDPA mesostructures such as micelles, micellar tubes, or condensed phases and their defined structural transitions could potentially be exploited in biotechnological applications or as drug delivery nanocarriers in biological environments. In this context, it is notable that pH-dependent phase transitions are sensitive to single amino acid mutations within the sequence. The width of the structural phase transition can be tuned by choosing hydrocarbon tails.

Furthermore, it is remarkable that permutations in the amino acid sequence led to different average conformations, e.g., extended or transient hairpin-like backfolding. Thus, disordered peptide motifs can result in distinctly different average conformations dependent on amino acid composition and sequence order. Last, we designed an enzymatically cleavable IDPA to demonstrate that IDPAs as surface-active components of nanocarriers can potentially react to metabolic conditions at target sites.

IDP-based headgroups may serve as grafted polymers for stabilizing particles via shell formation, as an alternative to poly(ethylene glycol) (PEG) lipids. Overall, their highly modular structure and function make IDPAs valuable to implement tailored functionalities and fine-tuned interactions for controllable structural phase transitions that could expedite cargo release. Based on our results and the discussed advantageous properties, we expect that IDPA conjugates will be valuable resources for the research community advancing precision medicine.

## Supporting Information Available

Chemical formulas, disorder analysis, FRET data, SAXS data, and details about SAXS analysis (PDF)
